# Efficacy of Bortezomib for Treating Anti-Interferon-Gamma Autoantibody-Associated Adult-Onset Immunodeficiency Syndrome

**DOI:** 10.1093/cid/ciad676

**Published:** 2023-11-08

**Authors:** Nasikarn Angkasekwinai, Yupin Suputtamongkol, Wiwit Tantibhedhyangkul, Nattawat Onlamoon, Pakpoom Phoompoung, Manop Pithukpakorn, Ekkapun Karuphong, Pawana Pusuwan, Pornpimon Angkasekwinai

**Affiliations:** Division of Infectious Diseases and Tropical Medicine, Department of Medicine, Faculty of Medicine Siriraj Hospital, Mahidol University, Bangkok, Thailand; Division of Infectious Diseases and Tropical Medicine, Department of Medicine, Faculty of Medicine Siriraj Hospital, Mahidol University, Bangkok, Thailand; Department of Immunology, Faculty of Medicine Siriraj Hospital, Mahidol University, Bangkok, Thailand; Research Group in Immunobiology and Therapeutic Sciences, Faculty of Medicine Siriraj Hospital, Mahidol University, Bangkok, Thailand; Division of Infectious Diseases and Tropical Medicine, Department of Medicine, Faculty of Medicine Siriraj Hospital, Mahidol University, Bangkok, Thailand; Division of Medical Genetics, Department of Medicine, Faculty of Medicine Siriraj Hospital, Mahidol University, Bangkok, Thailand; Division of Hematology, Department of Medicine, Faculty of Medicine Siriraj Hospital, Mahidol University, Bangkok, Thailand; Division of Nuclear Medicine, Department of Radiology, Faculty of Medicine Siriraj Hospital, Mahidol University, Bangkok, Thailand; Department of Medical Technology, Faculty of Allied Health Science, Thammasat University, Pathum Thani, Thailand

**Keywords:** efficacy, bortezomib, treating, anti-interferon-gamma autoantibody-associated adult-onset immunodeficiency (AOID) syndrome, opportunistic infection

## Abstract

**Background:**

Currently, there is no effective treatment for adult-onset immunodeficiency (AOID) syndrome with anti-interferon-gamma autoantibodies (anti-IFN-γ-auto-Abs). This study aimed to investigate the effectiveness of bortezomib (BTZ) for decreasing anti-IFN-γ-auto-Abs.

**Methods:**

A pre- and post-intervention study was conducted from February 2017 through June 2019 at Siriraj Hospital (Bangkok, Thailand). Five patients were invited to receive once-weekly BTZ (1.3 mg/m^2^ body surface area) subcutaneously for 8 weeks followed by oral cyclophosphamide (1 mg/kg/d) for 4 months. The primary outcomes were the difference in antibody level at 8 and 48 weeks compared with baseline and the incidence of serious adverse events (AEs). The secondary outcome was the occurrence of opportunistic infections (OIs) during the 72 weeks after starting BTZ.

**Results:**

The median patient age was 46 years (range, 34–53). All patients had 3–5 OIs prior to enrollment. All patients were receiving antimycobacterial agents for treatment of nontuberculous mycobacterial infection at enrollment. There was no significant difference in the mean optical density of auto-Abs at 8 weeks (3.73 ± 0.72) or 48 weeks (3.74 ± 0.53) compared with baseline (3.84 ± 0.49; *P* = .336 and *P* = .555, respectively). However, after serum dilution, the antibody titer nonsignificantly decreased 8–16 weeks after BTZ initiation (*P* = .345). Ten OIs were observed 24–72 weeks after BTZ initiation.

**Conclusions:**

Treatment with BTZ followed by cyclophosphamide yielded no significant decrease in antibody titer levels, and 10 OIs were observed during 24–72 weeks of BTZ treatment. No serious AEs were observed. Combining rituximab with BTZ is likely necessary to prevent generation of new autoantibody-producing plasma cells.

**Clinical Trials Registration**. NCT03103555.

## Teaser

Treatment with once-weekly bortezomib (BTZ) for 8 weeks followed by oral cyclophosphamide once daily for 4 months in 5 adult-onset immunodeficiency syndrome patients yielded no significant decrease in antibody titer levels. Ten opportunistic infections were observed 24–72 weeks after BTZ initiation.

The prevalence of adult-onset immunodeficiency (AOID) syndrome caused by anti-interferon-gamma autoantibodies (anti-IFN-γ-auto-Abs) is increasing worldwide, most predominantly in Asia [1–5]. Patients with anti-IFN-γ-auto-Abs commonly present with recurrent opportunistic infections (OIs), including nontuberculous mycobacteria (NTM) and other intracellular pathogens, such as *Salmonella* spp., varicella-zoster virus (VZV), *Cryptococcus* spp., *Histoplasma* spp., *Talaromyces* spp., and *Mycobacterium tuberculosis* [1, 2, 4]. The majority of patients have persistent infections that require prolonged oral antimycobacterial therapy. Some patients have recurrent infections (from prior or new pathogens) that require multiple courses of parenteral antimicrobial therapy [4–6]. Previous studies reported anti-IFN-γ-auto-Ab levels to be strongly associated with increased risk of OIs [1–4].

The anti-IFN-γ-auto-Ab level was also found to be strongly correlated with neutralizing and disease activities [4, 5]. There is currently no approved medication to reduce the auto-Ab level in this patient population. Rituximab (anti-CD20 monoclonal antibody) and intravenous cyclophosphamide were reported to reduce auto-Ab titers and to be significantly associated with clinical response and improved outcome [5, 7]. However, long-lived autoantibody-producing plasma cells are not targeted by rituximab.

Bortezomib (BTZ), which is a proteasome inhibitor that efficiently reduces both short-lived and long-lived plasma cells, was initially approved for the treatment of refractory multiple myeloma; however, it has also been studied in several autoantibody-mediated conditions, and it was found to reduce plasma cell and autoantibody production [8]. Our aim in this study was to investigate the effectiveness of BTZ for decreasing the level of anti-IFN-γ-auto-Abs and improving the clinical outcome of patients with AOID syndrome.

## METHODS

### Study Design

This pre- and post-intervention study was conducted from February 2017 through June 2019 at the Faculty of Medicine Siriraj Hospital, Mahidol University, Thailand's largest national tertiary referral center. The Siriraj Institutional Review Board approved the study protocol. Written informed consent to participate was obtained from all participants.

### Patients, Interventions, and Study Procedures

The inclusion criteria were aged ≥21 years, not diagnosed with human immunodeficiency virus (HIV), having positive anti-IFN-γ-auto-Abs, having a history of prior OIs, and having received antimicrobial therapy for treatment of previous OIs for at least 1 month before enrollment.

Eligible patients were invited to receive once-weekly BTZ subcutaneously at a dose of 1.3 mg/m^2^ body surface area for 8 weeks followed by oral cyclophosphamide at a dose of 1 mg/kg/d for 4 months. Patients were followed up once weekly for 8 weeks during the course of BTZ treatment, then every month until 24 weeks followed by every 2 months until 72 weeks after enrollment. Anti-IFN-γ-auto-Ab levels, laboratory parameters, immunoglobulin level, and flow cytometry were measured at preestablished time points. An F-18 fluorodeoxyglucose (F-18 FDG) positron emission tomography–computed tomography (PET–CT) whole-body scan was scheduled for baseline and 6 months to 1 year after enrollment. Adverse events (AEs) that occurred within 72 weeks of enrollment were also recorded. The study protocol, including scheduled interventions and procedures, is shown in Supplementary Table 1 and Supplementary Figures 1 and 2.

### Outcome Measurements

The primary outcomes were the difference in the level of anti-IFN-γ-auto-Ab titers (optical density [OD] and antibody level in arbitrary units [AUs]) at 8 weeks and 48 weeks compared with baseline and the proportion of serious AEs that occurred during the 72-week monitoring period. The secondary outcome was the occurrence of OIs, which was evaluated at 24 weeks, 48 weeks, and 72 weeks after BTZ initiation. An OI episode was defined as isolation of a culture-proven causative pathogen, including either a new pathogen or the same pathogen isolated previously. The one diagnostic exception was VZV, which was diagnosed clinically. Sample size calculation and statistical analysis information can be found in the Supplementary Material.

## RESULTS

The baseline characteristics of the 5 study patients are shown in Table 1. The median age of patients was 46 years (range, 34–53), and 3 of 5 patients were female. The duration of disease since diagnosis was 6, 3, 3, 4, and 4 years in patients 1 through 5, respectively, and 19 OIs were experienced among the 5 study patients prior to study enrollment. Rapidly growing mycobacteria, especially *Mycobacterium abscessus*, were the most common pathogens. All 5 patients were receiving antimycobacterial agents for treatment of NTM infection at the time of study enrollment.

**Table 1.** Baseline Characteristics of the 5 Study Patients

Characteristic	Case 1	Case 2	Case 3	Case 4	Case 5
Age, y	46	53	38	34	47
Gender	Female	Male	Female	Female	Male
Birthplace in Thailand	Sisaket Province	Nan Province	Roi Et Province	Saraburi Province	Nan Province
Comorbidity	Diabetes	None	None	None	None
Episodes of previous OIs	5	3	4	3	4
Types of previous OIs (specimen type[s], year of positive culture)	*Salmonella* spp. (blood and pus, 2011) *M. fortutitum* (blood, 2011) *Mycobacterium abscessus* (blood and LN, 2012) *Mycobacterium scrofulaceum* (blood, 2014; bone, 2016) *M. abscessus* (LN, 2016)	*Mycobacteria* spp. (LN, 2013; bone, 2014) *Talaromyces marneffei* (blood, 2016) *M. abscessus* (LN, 2017)	*Mycobacterium fortutitum* (LN, 2014) *Mycobacterium intracellulare* (blood, 2016) *Mycobacterium avium* complex (sputum, 2016) *M. abscessus* (blood, May and September 2017)	*Salmonella* spp. (blood, September and October 2013) *M. abscessus* (LN, 2014 and 2016) *M. abscessus* (tissue from left thigh, December 2017)	*Salmonella* spp. (blood, April, June, and July 2014) *M. abscessus* (LN, October 2017)
Reactive skin disease	No	Yes	Yes	Yes	Yes
Baseline laboratory investigations					
Antibody: optical density level	4.17	3.65	4.11	4.21	3.05
White blood cell count, cells/mm^3^	11 370	8370	35 340	13 420	7200
C-reactive protein, mg/L	34.69	1.68	6.69	26.14	1.65
Erythrocyte sedimentation rate, mm/h	82	8	56	56	4
Immunoglobulin level					
G	2970	1570	2240	2960	1800
A	377	236	201	381	243
M	84.9	128	162	183	128
E	527	639	67.3	1330	136
Immune cell count					
CD4 (%)	641 (12.97%)	550 (24.47%)	1242 (31.95%)	646 (31.07%)	1172 (37.91%)
CD8 (%)	1345 (27.24%)	554 (24.65%)	1191 (30.63%)	769 (36.97%)	613 (19.81%)
CD4/CD8	0.48	0.99	1.04	0.84	1.91
Date of first dose of BTZ	27 February 2017	8 May 2017	6 November 2017	25 December 2017	12 February 2018
Date of BTZ course completion	18 April 2017	26 June 2017	8 January 2018	12 February 2018	2 April 2018
Date of completion of all study drugs	21 August 2017	17 October 2017	4 May 2018	18 June 2018	6 August 2018

Abbreviations: BTZ, bortezomib; LN, lymph node; OI, opportunistic infection.

### Primary Outcomes: Anti-IFN-γ-Auto-Abs and the Proportion/Incidence of Serious AEs

**Figure 1.**
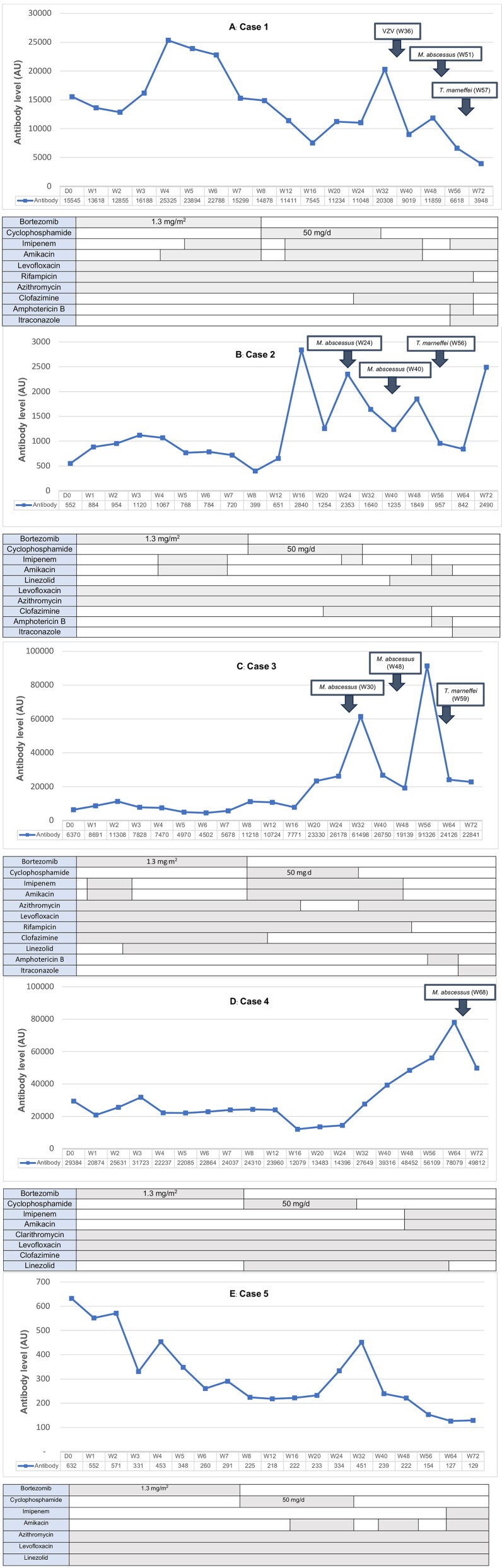
*A–E,* The levels of anti-interferon-gamma autoantibodies, the occurrence of opportunistic infections and the isolated causative pathogen, and the antimicrobial agents given from baseline to 72 weeks after the start of bortezomib treatment for each of the 5 study patients. Antibody levels (*y*-axis) are measured and reported in AUs. Abbreviations: AU, arbitrary unit; VZV, varicella-zoster virus.

The mean OD of anti-IFN-γ-auto-Abs among all patients at baseline was 3.84 ± 0.49. After BTZ initiation, the mean OD levels at 8 and 48 weeks were 3.73 ± 0.72 and 3.74 ± 0.53, respectively. No statistically significant difference was found between the OD level at 8 weeks (*P* = .336) or 48 weeks (*P* = .555) compared with baseline (Table 2). After diluting the serum and measuring the titers of autoantibody in AUs, the median antibody level was found to decrease during weeks 8–16 post-BTZ initiation. However, there was no significant difference in median antibody level between baseline (6370.1; interquartile range [IQR], 592.1–22 464.8) and 8 weeks (11 218.1; IQR, 311.6–19 593.7; *P* = .345) or 48 weeks (11 858.6; IQR, 1035.2–33 795.4; *P* = .345; Table 2). The levels of anti-IFN-γ auto-Abs, the occurrence of OIs and the causative pathogen, and the antimicrobial agents given from baseline to 72 weeks are shown in Figure 1*A*–*E*. Increased antibody level was also found to be associated with the timing of active infection as evidenced by increased C-reactive protein (CRP) and white blood cell (WBC) count levels. No serious AEs associated with BTZ treatment, such as leukopenia, thrombocytopenia, or neuropathy, were observed during the 72-week study period.

**Table 2.** Antibody Levels, Laboratory Parameters, and Proven Opportunistic Infections Within 72 weeks After Enrollment for Each of the 5 Study Patients

Primary Outcome: Antibody Level	Baseline	8 Weeks	24 Weeks	48 Weeks	72 Weeks
Patient 1					
OD level	4.17	4.27	3.79	4.13	4.10
AUs	15 545.3	14 877.6	11 047.7	11 858.6	3948.2
Other laboratory parameters					
WBC, cells/mm^3^	11.37	22 300	18 960	19 920	8280
CRP, mg/L	34.69	99.39	91.89	116.76	1.23
ESR, mm/h	82	81	102	119	60
Secondary outcome: proven OI(s)	Varicella-zoster virus infection: 36 wk after enrollment *Mycobacterium abscessus* (blood): 51 wk after enrollment *Talaromyces marneffei* (blood and LN): 57 wk after enrollment				
Patient 2					
OD level	3.65	4.03	3.81	3.2	3.97
AUs	551.9	1067.4	2353.4	1848.8	2489.7
Other laboratory parameters					
WBC, cells/mm^3^	8370	8990	30 560	23 870	7650
CRP, mg/L	1.68	1.31	109.35	88.25	2.17
ESR, mm/h	8	13	88	65	17
Secondary outcome: proven OI(s)	*M. abscessus* (LN [1+]): 24 wk after enrollment *M. abscessus* (LN [1 colony]): 40 wk after enrollment *T. marneffei* (synovial fluid left knee, bone): 56 wk after enrollment				
Patient 3					
OD level	4.11	4.01	4.13	4.12	4.05
AUs	6370	11 218.1	26 177.6	19 138.9	22 841.2
Other laboratory parameters					
WBC, cells/mm^3^	35 340	16 850	11 800	28 130	32 520
CRP, mg/L	6.69	15.12	1.47	35.91	191.05
ESR, mm/h	56	51	49	69	17
Secondary outcome: proven OI(s)	*M. abscessus* (blood): 30 wk after enrollment *M. abscessus* (blood): 48 wk after enrollment *T. marneffei* (right supraclavicular LN): 59 wk after enrollment				
Patient 4					
OD level	4.21	3.88	4.10	4.12	4.16
AUs	29 384.4	24 309.7	14 396.2	48 451.8	49 811.5
Other laboratory parameters					
WBC, cells/mm^3^	13 420	17 410	17 310	15 660	7890
CRP, mg/L	26.14	25.63	28.53	20.73	6.67
ESR, mm/h	56	50	88	95	59
Secondary outcome: proven OI(s)	*M. abscessus* (LN): 68 wk after enrollment				
Patient 5					
OD level	3.05	2.46	3.42	3.12	2.47
AUs	632.1	224.6	333.5	221.6	129.3
Other laboratory parameters					
WBC, cells/mm^3^	7200	8090	7990	11 500	7640
CRP, mg/L	1.65	2.47	8.31	15.85	3.08
ESR, mm/h	4	1	4	8	5
Secondary outcome: proven OI(s)	No proven OI(s) after study enrollment				

Abbreviations: AU, arbitrary unit; CRP, C-reactive protein; ESR, erythrocyte sedimentation rate; LN, lymph node; OD, optical density; OI, opportunistic infection; WBC, white blood cell.

### Secondary Outcome: Occurrence of OIs During the Follow-up Period

The type and timing of OIs among the 5 study patients are shown in Table 2 and Figure 1*A*–*E*. Four of 5 patients had a total of 10 OI episodes within 72 weeks after starting BTZ treatment for a mean of 1.33 episodes/patient/year (range, 0–4). All OIs occurred 24–72 weeks after BTZ initiation. The mean time from BTZ initiation to the occurrence of OIs was 39.5 weeks (range, 24–68). Among the 10 aforementioned OI episodes, *M. abscessus* was the most common causative pathogen followed by *Talaromyces marneffei* and herpes zoster virus infection. The number of OIs, the number of hospitalizations, and the total hospital length of stay compared between 72 weeks before and 72 weeks after enrollment are shown in Supplementary Table 2.

### Characterization of B- and T-Cell Subsets

**Figure 2.**
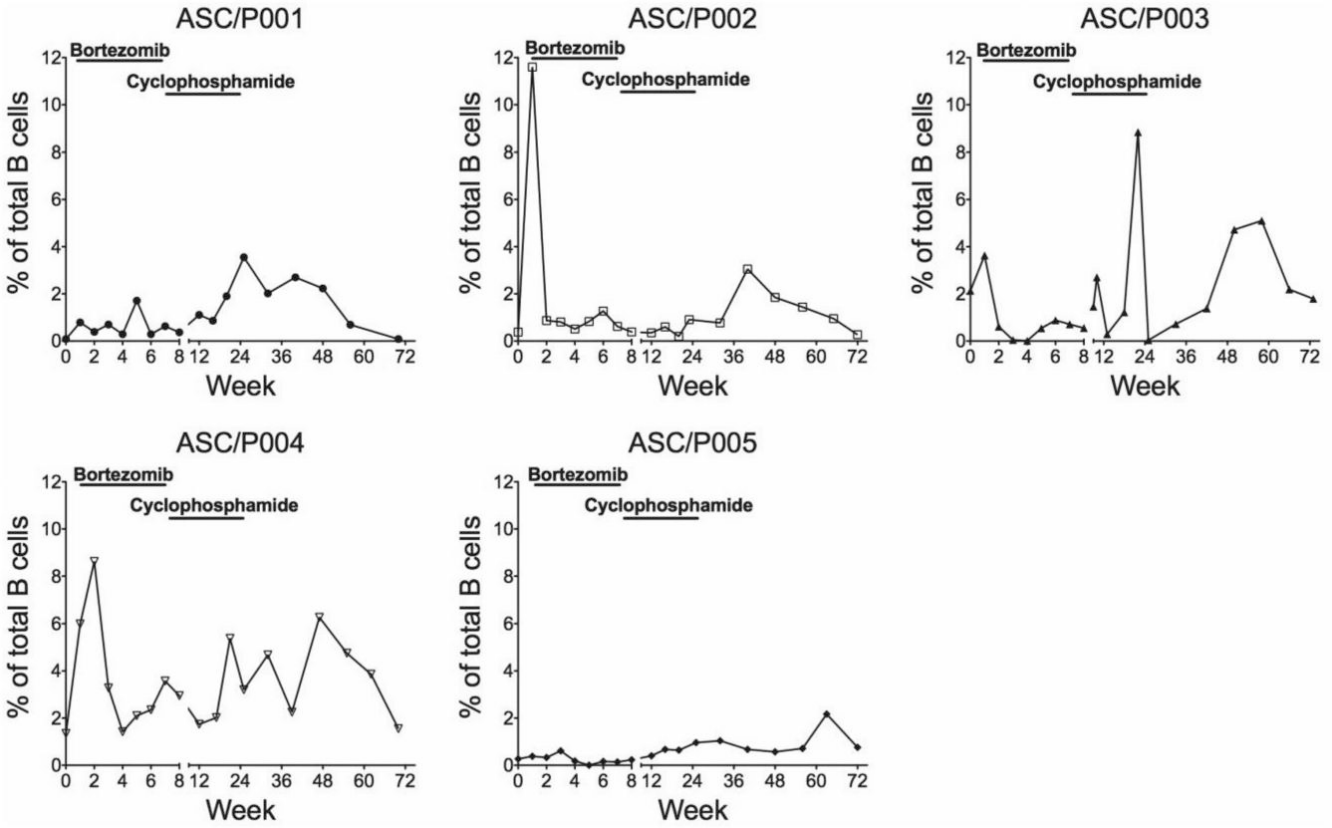
Kinetic analysis of antibody-secreting cells for each of the 5 study patients. The percentages of antibody-secreting cells among B-cell populations were determined at different time points during the course of the study. Abbreviation: ASC, antibody-secreting cell.

Antibody-secreting cells (ASCs) are responsible for autoantibody production against IFN-γ, and kinetic analysis of this cell population is shown in Figure 2. Among the 5 patients enrolled in this study, 3 exhibited marked alterations in ASCs during BTZ treatment (P002, P003, and P004). The results showed an increased percentage of ASCs during weeks 1 and 2, followed by a considerably decreased level for the remainder of the study period. However, fluctuations in ASC levels were observed during the 72-week study period in all patients.

For T-cell analysis, alterations in activated T cells were observed, and the results showed high percentages of activated CD4+ and CD8+ T cells throughout the 72-week study period (Supplementary Figure 3). Cell populations with the CD38-DR+ phenotype were comparatively stable, whereas high fluctuations were observed for the CD38+DR+ phenotype.

### Suppression of IFN-γ Function by Autoantibody

**Figure 3.**
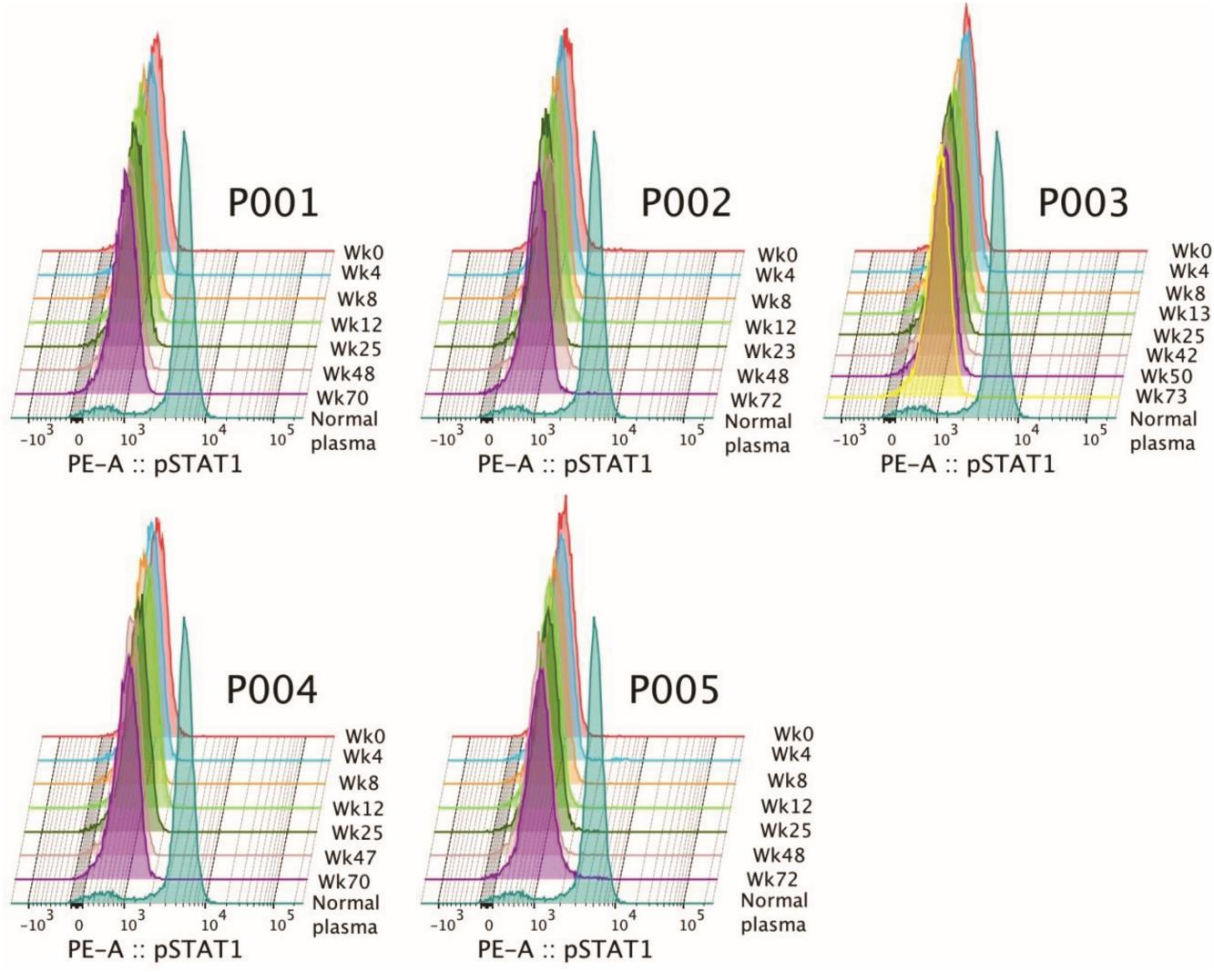
Inhibition of phospho-STAT-1 induction. The ability of plasma samples to inhibit phosphorylation of the STAT-1 protein was compared between different time points during the course of the study. Plasma samples from healthy individuals were used as the control (normal plasma).

Since IFN-γ can induce phosphorylation of the STAT-1 (pSTAT-1) protein in monocytes, the presence of autoantibody against IFN-γ could inhibit the induction of pSTAT-1, which can be observed by flow cytometric analysis. IFN-γ-induced pSTAT1 was observed when peripheral blood mononuclear cells (PBMCs) were incubated with plasma from healthy individuals; however, its fluorescence intensity decreased when PBMCs were incubated with plasma from AOID patients (Figure 3). Although plasma samples from all patients were shown to inhibit pSTAT-1 induction, no significant difference in the fluorescence intensity of pSTAT-1 was observed when plasma samples from different time points from a single patient were used.

To investigate alterations in autoantibody levels in plasma samples after BTZ treatment, an increased concentration of IFN-γ (100 ng/mL) was used. The results showed that the levels of autoantibody in plasma at weeks 0, 8, and 12 were too low to suppress the induction of pSTAT-1 when a high concentration of IFN-γ was used (Supplementary Figure 4).

### F-18 FDG PET–CT Whole-Body Scan

**Figure 4.**
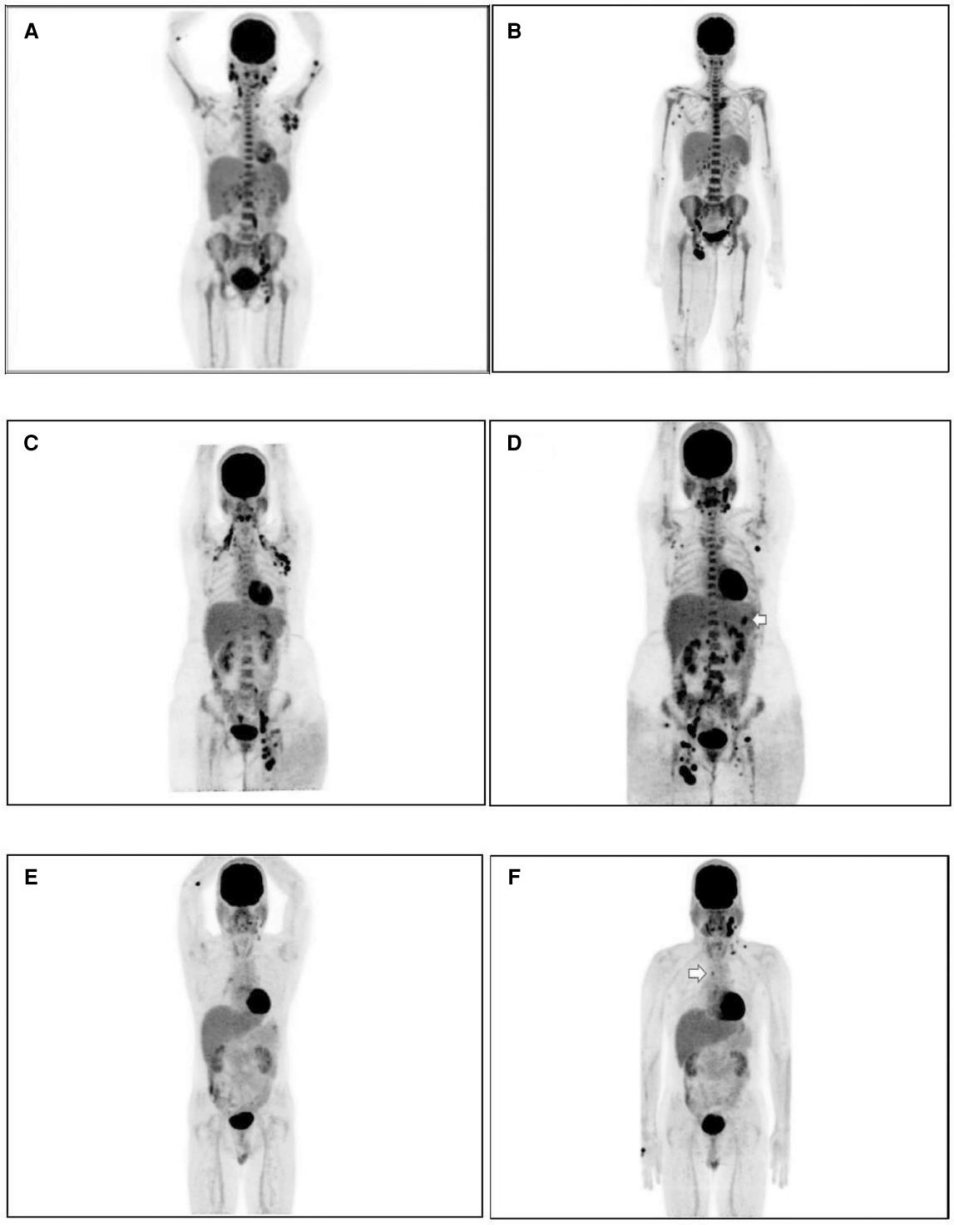
Pretreatment and follow-up whole-body F-18 fluorodeoxyglucose positron emission tomography–computed tomography scans of patients 3, 4, and 5. *A,* Pretreatment scan of patient 3 showed increased uptake in bilateral cervical nodes, bilateral supraclavicular nodes, bilateral axillary nodes, intraabdominal nodes, and left iliac nodes; hepatosplenomegaly; and generalized increased uptake in the axial spine and proximal bones of all extremities. *B,* Repeat scan after 2 years of bortezomib (BTZ) therapy revealed decreased size and uptake in left cervical nodes, left axillary nodes, intraabdominal nodes, and left iliac nodes. New areas of increased uptake were observed in right axillary nodes, right hilar nodes, right iliac nodes, and right inguinal nodes. Slightly increased uptake and generalized edema of the right thigh was also found. Unchanged hepatosplenomegaly and generalized increased uptake was seen in the axial spine and proximal bones of all extremities. *C,* Pretreatment scan of patient 4 showed increased uptake in bilateral cervical nodes, bilateral axillary nodes, pelvic nodes, and bilateral inguinal nodes with skin thickening and generalized increased uptake at the proximal left thigh. *D,* Repeat scan after 1 year of BTZ therapy revealed decreased number and size of lymph nodes at the cervical and left inguinal regions, but new areas of increased uptake were observed, including in 1 node at the splenic hilum (arrow) and in para-aortic nodes, pelvic nodes, and multiple right inguinal nodes. Progression of bilateral skin thickening with slightly increased uptake at the proximal bilateral thighs was also found. *E,* Pretreatment scan of patient 5 showed increased uptake in left cervical nodes and in a right hilar node. *F,* Repeat scan after 1 year of BTZ therapy revealed increased size, number, and uptake in left cervical nodes, left supraclavicular nodes, and in 1 right lower paratracheal node (arrow).

All patients had lymphadenopathies at multiple locations at baseline that appeared to persist or progress at 6 months to 1 year after initiation of BTZ. Despite the PET–CT scans of patients 3 and 4 showing decreased size of some lymphadenopathies, new locations of hypermetabolic lymphadenitis were also found. No OIs developed in patient 5 during the follow-up; however, that patient's PET–CT scan at 48 weeks still showed the overall progression of active disease. Figure 4 shows the F-18 FDG PET–CT whole-body scans of patients 3, 4, and 5 at baseline compared with the follow-up period. The results of F-18 PET–CT whole-body scan at baseline and during the follow-up period for all 5 patients are summarized in Supplementary Table 3.

## DISCUSSION

Currently, there is no approved immunomodulatory agent to reduce anti-IFN-γ-auto-Abs in patients with AOID syndrome. Patients with high titers of anti-IFN-γ-auto-Abs are recurrently infected with intracellular pathogens. BTZ, which is a proteasome inhibitor that was approved for treatment of multiple myeloma, was demonstrated in animal studies to kill short-lived and long-lived plasma cells [9], which is the main antibody-producing cell type, and was found to reduce autoantibodies in several autoimmune conditions, including myasthenia gravis, systemic lupus erythematosus (SLE), chronic allograft nephropathy, and antibody-mediated kidney transplant rejection [10–13].

This pilot study showed that administration of once-weekly BTZ for 8 weeks followed by oral cyclophosphamide for 4 months in 5 patients with high titers of anti-IFN-γ-auto-Abs could not influence a significant decrease in OD level at 8 weeks or 48 weeks compared with baseline when using a standard method (serum dilution, 1:100). After further diluting the serum of each patient (measured in AUs), a decrease in Ab titer was observed in all patients. The aforementioned change in results is most likely due to signal saturation when using the standard procedure. Consequently, it can be postulated that the observed change in Ab titers can be attributed to application of a dilution factor. Antibody titers are more sensitive to detection after serum dilution when compared with detection via the routine method. Fluctuations in antibody concentrations were observed during the 72-week study period. The half-life of BTZ was reported to vary from 144 hours to 23 days [14, 15]. The use of repeated cycles of BTZ to achieve sustained antibody reduction requires further study. However, it has been postulated that an effective antimicrobial treatment for infection can also play an important role in decreasing antibody levels. Therefore, this factor must be considered when assessing the response to any immunosuppressive agents during disease monitoring. This study also suggests the use of WBC count and CRP level as biomarkers for monitoring active infection in this patient population, which is similar to previous studies [4, 16].

No recurrent OIs developed within 6 months after the start of BTZ therapy; however, 4 of 5 patients developed a total of 10 recurrent OIs over 24–72 weeks, and the mean duration of OI was approximately 10 months. The incidence of OIs compared between 72 weeks before BTZ treatment (9 episodes) and 72 weeks after BTZ treatment (10 episodes) was very similar. The finding that *M. abscessus* was the most frequent causative pathogen among patients with anti-IFN-γ-auto-Abs is similar to the results reported from prior studies conducted in Asia [3, 16, 17]. *Talaromyces marneffei* occurred in 3 of 5 patients after BTZ treatment. From our previous study, *T. marneffei* infection in patients with anti-IFN-γ-auto-Abs at our center was observed in 9 of 80 patients (11.3%), or 9 of 194 total OI episodes (5.0%), which is significantly lower than the OI incidence among those who received BTZ in the present study [4]. However, it should be noted that the clinical characteristics of the 80 patients in our previous study varied from active or persistent disease to drug-free remission. The occurrence of OIs after BTZ treatment should be attributed primarily to AOID with anti-IFN-γ-auto-Abs rather than to immunodeficiency caused by BTZ treatment. A previous study in SLE patients found that peripheral blood plasma cells quickly regenerated within 10 days after the last dose of BTZ [18]. BTZ also exerts potent immunosuppressive effects on T cells and was reported to increase the risk of herpes simplex and zoster virus reactivation [19]. In the present study, we found 1 episode of VZV among our 5 study patients that could have been due to AOID with anti-IFN-γ-auto-Abs or infection-related therapy. In contrast, we did not monitor nonserious AEs, such as mild infection, nausea, or vomiting, that may be related to BTZ in this study. A longer duration of follow-up in a larger BTZ-treated study sample will be needed to bring added clarity to the aforementioned unknowns.

The presence of autoantibody against IFN-γ during NTM infections is well established; however, the involvement of ASCs in the maintenance of anti-IFN-γ-auto-Ab serum levels in AOID syndrome patients has never been addressed. Recent studies demonstrated the presence of a cell subset with CD19+CD20-CD27+CD38+ phenotype that has the ability to secret antibody, either in peripheral blood or in cerebrospinal fluid, in patients with autoimmune disorders, such as neuromyelitis optica spectrum disorder and multiple sclerosis [20, 21]. Therefore, the level of ASCs was determined in this study to observe for potential alteration of this B-cell subset after administration of BTZ. The results showed alteration of ASCs in our study patients after BTZ treatment; however, the ASC levels were inconsistent during the course of the study. Variations in ASC levels may be dependent on whether the patient was in a stable condition or had active disease at the evaluation time point. Since it is unclear whether anti-IFN-γ antibody or pathogen-specific antibody was produced by these ASCs, further study is needed to determine the type of antibody produced and to determine whether their production capacity is altered after BTZ treatment.

Since T cells play an important role during mycobacterial infections, the levels of activated T cells and their alteration after BTZ treatment were observed in this study. The expressions of CD38 and human leukocyte antigen-DR (HLA-DR) were used to assess the level of T-cell activation in both acute and chronic infections, such as dengue and HIV [22, 23], and in *M. tuberculosis* infection [24]. In this study, our results showed markedly increased activated T cells that coexpress CD38 and HLA-DR. Despite high levels of activated T cells being observed, their specific role remains unclear. A recent study suggested that the presence of anti-IFN-γ-auto-Abs in serum inhibited CD4+ Th1 and CD8+ T-cell immune responses [25]. It is possible that consistently high levels of activated T cells persisted in our patients due to the reduction in anti-IFN-γ-auto-Ab levels following BTZ treatment.

The results of our study also confirmed that the presence of anti-IFN-γ-auto-Abs has the ability to suppress IFN-γ-induced STAT1 phosphorylation [26]. A previous study demonstrated that plasma from patients who had been treated with rituximab showed inhibition of IFN-γ-induced pSTAT-1 production [27]. Although the results of our study showed that plasma samples from patients after BTZ administration had preserved ability to suppress IFN-γ-induced STAT1 phosphorylation, decreased intensity of pSTAT-1 was observed when a high concentration of IFN-γ was used, which suggests that BTZ treatment partially reduced anti-IFN-γ-auto-Ab levels.

F-18 FDG PET–CT has been widely used for noninvasive imaging, staging, and monitoring of treatment response in patients with neoplastic diseases [28, 29] and has also been used to diagnose or monitor response to treatment in several infectious conditions [30]. This is the first study to use the F-18 FDG PET–CT whole-body scan to monitor active disease in patients with AOID syndrome with anti-IFN-γ-auto-Abs. We found that all patients had extensive hypermetabolic lymphadenopathies at baseline, not only in superficial lymph nodes but also in intrathoracic and intraabdominal lymph nodes. Second, we observed independent response among different lesion sites within the same patient over time, and we observed both regression and progression of different lesions in the follow-up PET–CT study. This finding is consistent with the results from a previous study in tuberculosis patients that reported heterogeneous response after anti-tuberculosis treatment [30,31–32]. Overall, we found the F-18 FDG PET–CT whole-body scan to be remarkably helpful in assessing the extent of lymphadenopathies at both baseline and during treatment in this study. Importantly, interpretation of F-18 FDG uptake should be performed with caution since interpretation of F-18 FDG uptake alone in the absence of clinical disease may reflect host response to infection rather than active infection.

Despite the ability of BTZ to rapidly decrease the concentration of short-lived and long-lived plasma cells in both bone marrow and peripheral blood, it has little effect on plasma cell progenitors. A previous study of BTZ in SLE patients reported autoantibody reproduction after BTZ withdrawal [33]. This phenomenon was evidenced by a rapid increase in circulating plasma cells in peripheral blood, which suggests that the inhibitory response exerted by BTZ on autoantibody production is transient. Therefore, combining BTZ with an immunosuppressive agent that targets plasma cell precursors, such as rituximab, is likely necessary to prevent the generation of new plasma cells. A previously published case study reported successful reduction of anti-IFN-γ-auto-Abs and improved IFN-γ-induced STAT-1 phosphorylation after BTZ administration in patients with refractory infections after rituximab treatment, which resulted in a better clinical and radiological outcome [34]. In addition, daratumumab, which is a novel anti-CD38 monoclonal antibody, was also reported to decrease anti-IFN-γ-auto-Abs after rituximab combined with BTZ [35].

Despite the fact that our study sample satisfied the minimum established by our sample size calculation, the notable limitation of this study is its small sample size. The fact that we enrolled only 5 patients suggests that our results may not be generalizable to all patients with AOID associated with anti-IFN-γ-auto-Abs.

## CONCLUSIONS

Treatment with BTZ followed by cyclophosphamide yielded no significant decrease in antibody titer levels. Ten OIs were observed during 24–72 weeks, and *M. abscessus* was the most prevalent causative organism. WBC count, CRP, and F-18 FDG PET–CT whole-body scan were all found to be useful in assessing patient response to treatment. Combining rituximab with BTZ is likely necessary to prevent generation of new autoantibody-producing plasma cells.

## Supplementary Data

Supplementary materials are available at *Clinical Infectious Diseases* online. Consisting of data provided by the authors to benefit the reader, the posted materials are not copyedited and are the sole responsibility of the authors, so questions or comments should be addressed to the corresponding author.

## Supplementary Material

ciad676_Supplementary_Data

## Notes

***Acknowledgments.*** We gratefully acknowledge the 5 patients who generously agreed to participate in this study and Ekarat Wongsawat for study coordination.

***Author contributions.*** Study conception and design: N. A., Y. S., W. T., N. O., P. P., M. P., and E. K.; data collection: N. A., W. T., N. O., P. P., and E. K.; analysis and interpretation of results: N. A., Y. S., W. T., N. O., P. P., M. P., E. K., P. P., and P. A.; draft manuscript preparation: N. A., Y. S., W. T., N. O., P. P., M. P., E. K., P. P., and P. A. All authors reviewed the results and approved the final version of the manuscript to be submitted for journal publication.

***Disclaimer.*** The funding source had no role in the study design; in data collection, analysis, or interpretation; in the conclusions drawn; in the preparation of the manuscript; or in the decision to submit the completed manuscript for journal publication.

***Data sharing statement.*** Additional data is available in the supplementary material. Requests for additional data should be directed to the corresponding author.

***Financial support*.** This study was supported by a grant from the Health Systems Research Institute, Nonthaburi, Thailand (grant 61-049).
