# Supplementary material for: Efficacy of Bortezomib for Treating Anti-Interferon-Gamma Autoantibody-Associated Adult-Onset Immunodeficiency Syndrome
Source: Clin Infect Dis. 2023 Nov 8;78(4):1033–42. doi: 10.1093/cid/ciad676 (PMC11006116; doi:10.1093/cid/ciad676)
Supplement: ciad676_Supplementary_Data [file ciad676_supplementary_data.docx]

**Supplementary material**

**Study protocol**

1. **Patients**

The inclusion criteria were age ≥21 years, human immunodeficiency virus (HIV)-negative, having positive anti-IFN-γ-auto-Abs, history of prior OIs, and history of having received antimicrobial therapy for treatment of previous OIs for at least one month. Patients with/having one or more of the following were excluded: pregnant or lactating women; receiving immunosuppressive therapy for other chronic disorders; absolute neutrophil count <1,500 cells/µL, platelets <150,000/ µL, or hemoglobin level <8 g/dL; acute respiratory distress syndrome (ARDS); elevated total bilirubin >1.5 times or aspartate transaminase (AST) >3 times the upper normal limit (UNL); end-stage renal disease (ESRD) with an estimated glomerular filtration rate (eGFR) <30 ml/minute or requiring renal replacement therapy; and/or, history of hypersensitivity to BTZ.

1. **Interventions and study procedures**

After written informed consent was obtained, an interview was conducted to collect and record patient baseline medical history, and physical examinations and baseline laboratory investigations were performed. Eligible patients were invited to receive once weekly BTZ subcutaneously at a dose of 1.3 mg/m^2^ body surface area (BSA) for 8 weeks, and oral cyclophosphamide at a dose of 1 mg/kg/day for 4 months. For this study, we selected the standard dose of bortezomib (1.3 mg/m^2^) that is used to treat multiple myeloma (1). Concerning oral cyclophosphamide dosing, a dose of 1-2 mg/kg is routinely used to treat several autoimmune diseases. For this study, we selected the fixed lowest dose of 50 mg/day. Cyclophosphamide is known to suppress B cell function and antibody production (2); therefore, we hypothesized that combination cyclophosphamide and BTZ may effectively control autoantibody production in patients with AOID. *In vitro* studies reported the synergistic effects of combination cyclophosphamide and BTZ in myeloma cells, which are abnormal plasma cells that influence antibody production (3-4). Moreover, combination bortezomib and cyclophosphamide has been used to treat multiple myeloma, and the side effects of this combination regimen were found to be tolerable (5).

Patients were followed-up once weekly for 8 weeks during the course of BTZ, and then every month until 24 weeks followed by every two months until 72 weeks after enrollment.

Supplementary Table 1 summarizes the study procedures that were performed at various steps/weeks during the 72-week period after the initiation of BTZ treatment. The investigations performed at baseline and at each follow-up visit throughout 72 weeks included complete blood count (CBC); inflammatory biomarker levels, including C-reactive protein (CRP) and erythrocyte sedimentation rate (ESR); and, anti-IFN-γ-auto-Ab levels. Immunoglobulin G (IgG), cluster of differentiation (CD) 4 level, CD8 level, and flow cytometry were measured at baseline, 4 weeks, and 12 weeks after BTZ initiation. An F-18 fluorodeoxyglucose (F-18 FDG) positron emission tomography-computed tomography (PET/CT) whole body scan was scheduled for baseline and 6 months to one year after study enrollment (Supplementary Table 2). For each OI that developed during the follow-up period, clinical specimens, such as blood culture and lymph node aspiration and/or biopsy, were obtained to identify the causative pathogen(s). Other laboratory investigations were performed as clinically indicated. Adverse events that occurred within 72 weeks of enrollment were also collected and recorded. All data were recorded on standardized case record forms.

1. **Laboratory methods**

***Indirect ELISA for anti-IFN-γ autoantibody detection***

Anti-IFN-γ-auto-Ab concentrations in serum were measured using an enzyme-linked immunosorbent assay (ELISA), which is the same method that was used in a previously published study by our research group.^4^ Briefly, serum was 100-fold diluted with phosphate-buffered saline-0.1% Tween120 (PBST) containing 1% bovine serum albumin (BSA). A volume of 100 μL of diluted sera was transferred to a 96-well ELISA plate (Corning, Inc., Corning, NY, USA) coated with 0.5 μg/ml of recombinant human IFN-γ (R&D Systems, Minneapolis, MN, USA) and incubated at room temperature (RT) for one hour. After washing, IFN-γ autoantibodies were detected using alkaline phosphatase-conjugated goat anti-human IgG antibody (Southern Biotech, Birmingham, AL, USA) at 1:2,000 dilution with PBST-1% BSA. After washing, color was developed using *para*-nitrophenyl phosphate (*p*-NPP) and the absorbance was measured using 405 nm detection and a 630 nm reference wavelength. The relative concentration of anti-IFN-γ-auto-Abs is described as the optical density (OD) of a patient sample compared to that of a healthy control, and an OD of greater than 1 was considered to be a positive result.

***Quantitative measurement of anti-IFN-γ autoantibody level***

First, we generated a standard curve to quantify the level of serum autoantibody. Briefly, the serum from every visit (19 visits of each patient) of all 5 study patients, given a total of 95 specimens was pooled and two-fold diluted (from 1:100 to 1:1,638,400) to obtain 15 dilutions. Using open-source software (https://www.myassays.com/), four-parameter logistic (4PL) regression was used to construct the standard curve. The autoantibody level at a serum dilution of 1:12,800 (half of the measurable range) was defined as one arbitrary unit (AU). The obtained standard curve was used to determine the autoantibody level in pooled serum prepared from different visits by each patient. The autoantibody level in each pooled serum specimen was calculated using the first dilution factor at which the OD exceeded 1. No unit of measurement was used for optical density (OD) since a numeric value alone is routinely used. We then used pooled serum from every visit of each individual patient to generate the second standard curve to be used for that specific patient (Supplementary Figure 1). The serum obtained from each patient at each follow-up visit was diluted to the proper concentration and measured using ELISA. The auto-Ab concentration was calculated using the following formula: AU × dilution factor = auto-Ab concentration.

***Determination of B cell subsets and activated T cells by flow cytometric analysis***

The heparinized whole blood samples were stained with a combination of fluorochrome-conjugated monoclonal antibodies (mAbs) against CD3, CD4, CD8, CD14, CD19, CD20, CD21, CD27, CD38, CD45, and HLA-DR for B and T cell subset analysis. After incubation for 15 minutes, the red blood cells were lysed using red blood cell lysis solution. Cell pellets were then obtained by centrifugation at 450 times gravity (x g) for 5 minutes followed by washing with phosphate buffered saline (PBS). The stained samples were resuspended with PBS and maintained at 4 degrees Celsius (°C) until flow cytometric analysis using a BD LSRFortessa™ Cell Analyzer with FACSDiva software (both BD Biosciences, San Jose, CA, USA). Data analysis was performed using FlowJo software (BD Biosciences).

***Detection of phospho-STAT-1 induction by flow cytometry***

Phosphorylated signal transducer and activator of transcription (pSTAT-1) expression was determined via flow cytometric analysis. Peripheral blood mononuclear cells (PBMCs) from healthy donors were resuspended in complete Roswell Park Memorial Institute (RPMI) media at a concentration of 1 x 10^6^ cells/800 µL. Whole blood samples from one healthy volunteer were obtained for isolation of PBMCs. Cell suspensions were mixed with 100 µL of plasma sample from a study patient for 10 minutes followed by the addition of 1 and 100 ng/mL of recombinant human interferon gamma (rhIFN-γ) for 15 minutes. Intracellular staining with PE anti-STAT1 Phospho (Tyr701) Antibody (BioLegend, San Diego, CA, USA) was performed according to the manufacturer’s instructions. Stained samples were maintained at 4°C until flow cytometric analysis using a BD LSRFortessa™ Cell Analyzer with FACSDiva software (both BD Biosciences). Data analysis was performed using FlowJo software (BD Biosciences).

Antibody-secreting cells (ASCs) were defined as a cell population that expresses a high density of CD38 in the absence of CD20 (the gating strategy that was used for B cell subset analysis is shown in Supplementary Figure 1). B cells (CD20+ cells) were further subcategorized according to their expression of CD21 and CD27, including naïve (CD21+CD27-), resting memory (CD21+CD27+), activated memory (CD21-CD27+), and tissue memory (CD21-CD27-) cells. For T cell analysis, T cell activation based on the expression of CD38 and the major histocompatibility complex, class II, DR alpha (HLA-DR) gene was determined (Supplementary Figure 2).

1. **F-18 FDG PET/CT whole body scan**

All patients were scheduled for an F-18 fluorodeoxyglucose (FDG) positron emission tomography-computed tomography (PET/CT) scan at baseline and at 6 months to one year after study enrollment. Four of the 5 study patients had the follow-up scan within the prescribed window (2 at six months, and 2 at 1 year); however, 1 patient (patient 3) failed to show for the scheduled 1-year follow-up scan and ended up being scanned at 2 years after the start of BTZ treatment. Imaging was performed using a Discovery PET/CT system (GE Healthcare, Chicago, IL, USA) 60 minutes after intravenous injection of 10-15 mCi F-18 FDG. Low-dose CT acquisition was performed first using the following parameters: 140 kV, 80 mA, 0.8 seconds per CT rotation, pitch of 6, and table speed of 22.5 mm/second. A PET emission scan was performed immediately after acquisition of the CT images with no change in patient positioning. Five to 7 bed positions were performed from vertex to midthigh. PET images were reconstructed using an ordered subset expectation maximization algorithm. CT data were used for attenuation correction. Studies were interpreted on a Xeleris™ Workstation (GE Healthcare). Regarding image interpretation, whole body F-18 FDG PET/CT scans were interpreted by consensus reached between a nuclear medicine physician and a radiologist. An abnormal PET scan was defined as an area of increased F-18 FDG uptake greater than that of the surrounding background and that cannot be explained by normal organ uptake.

1. **Outcome measurements**

The primary outcomes were the difference in the level of anti-IFN-γ-auto-Ab titers (optical density and antibody level in AUs) at 8 weeks and 48 weeks compared to baseline, and the proportion of serious adverse events (SAEs) that occurred within the 72-week monitoring period. We defined an SAE as any event that resulted in death, that was life-threatening, that required hospitalization, that resulted in persistent or substantial disability, or that had important medical consequences. However, a hospitalization for intravenous antimicrobial therapy could occur due to the natural history of AOID, so these events were not considered to be SAEs in this study. BTZ-specific SAEs, including grade 3 or greater neutropenia, thrombocytopenia, or neuropathy, were monitored for, collected, and recorded; however, BTZ-specific non-SAEs were not monitored for in this study. The secondary outcome was the occurrence of OIs, which was evaluated at 24 weeks, 48 weeks, and 72 weeks after the start of BTZ treatment. An OI episode in this study was defined as isolation of a culture proven causative pathogen, including either a new pathogen or the same pathogen isolated previously. The one diagnostic exception was the varicella zoster virus (VZV), which was diagnosed clinically. Any worsening of clinical manifestation without a positive culture was not counted as a new OI.

1. **Sample size calculation and statistical analysis**

We calculated the sample size to estimate a single group mean with a specified level of confidence and precision. From a previous unpublished study, the mean anti-IFN-γ-auto-Ab titer level of patients during active disease was 3.97 (95%CI: 3.84-4.09). Using a 95% confidence level, a 15% level of precision for estimating a mean value of 3.97, and a standard deviation of 0.6, the required sample size to estimate the single group mean was 5 patients.

The data were analyzed using SPSS Statistics for Windows, version 18.0 (SPSS, Inc., Chicago, IL, USA). Categorical data are presented as number or number and percentage. Normally and non-normally distributed continuous data are presented as mean plus/minus standard deviation (SD) and as median and range or interquartile range (IQR), respectively. Friedman test was used to evaluate changes in anti-IFN-γ auto-Ab levels over time. A *p*-value less than 0.05 was regarded as being statistically significant for all tests.

**References**

1. Vincent Rajkumar S. Multiple myeloma: 2014 Update on diagnosis, risk-stratification, and management. Am J Hematol. 2014 Oct;89(10):999-1009.
2. Hofmann K, Clauder AK, Manz RA. Targeting B Cells and Plasma Cells in Autoimmune Diseases. Front Immunol. 2018;9:835.
3. Ma MH, Yang HH, Parker K, et al. The proteasome inhibitor PS-341 markedly enhances sensitivity of multiple myeloma tumor cells to chemotherapeutic agents. Clin Cancer Res. 2003;9:1136–1144.
4. Mitsiades N, Mitsiades CS, Richardson PG, et al. The proteasome inhibitor PS-341 potentiates sensitivity of multiple myeloma cells to conventional chemotherapeutic agents. Blood. 2003;101:2377–2380.
5. Kapoor P, Ramakrishnan V, Rajkumar SV. Bortezomib combination therapy in multiple myeloma. Semin Hematol. 2012 Jul;49(3):228-42.

**Supplementary Table 1.** Study procedures that were performed at various steps/weeks during the 72-week period after the initiation of bortezomib treatment

| **Procedures** | **Screening** | **D0** | **W1** | **W2** | **W3** | **W4** | **W5** | **W6** | **W7** | **W8** | **W12** | **W16** | **W20** | **W24** | **W32** | **W40** | **W48** | **W56** | **W64** | **W72** |
| --- | --- | --- | --- | --- | --- | --- | --- | --- | --- | --- | --- | --- | --- | --- | --- | --- | --- | --- | --- | --- |
| Informed consent obtained | x |  |  |  |  |  |  |  |  |  |  |  |  |  |  |  |  |  |  |  |
| Review for study eligibility | x |  |  |  |  |  |  |  |  |  |  |  |  |  |  |  |  |  |  |  |
| Symptoms & physical exam | x | x | x | x | x | x | x | x | x | x | x | x | x | x | x | x | x | x | x | x |
| Assess adverse events | x | x | x | x | x | x | x | x | x | x | x | x | x | x | x | x | x | x | x | x |
| Laboratory investigation |  |  |  |  |  |  |  |  |  |  |  |  |  |  |  |  |  |  |  |  |
| Urine pregnancy test ^a^ | x | x |  |  |  | x |  |  |  | x | x | x | x | x |  |  |  |  |  |  |
| Anti-HIV ^b^ | x |  |  |  |  |  |  |  |  |  |  |  |  |  |  |  |  |  |  |  |
| Electrocardiogram | x |  |  |  |  |  |  |  |  |  |  |  |  |  |  |  |  |  |  |  |
| Chest X-ray | x |  |  |  |  |  |  |  |  |  |  |  |  |  |  |  |  |  |  |  |
| Complete blood count | x | x | x | x | x | x | x | x | x | x | x | x | x | x | x | x | x | x | x | x |
| Biomarker: CRP/ESR | x | x | x | x | x | x | x | x | x | x | x | x | x | x | x | x | x | x | x | x |
| IFN-gamma autoantibodies | x | x | x | x | x | x | x | x | x | x | x | x | x | x | x | x | x | x | x | x |
| Blood chemistry ^c^ | x | x |  |  |  | x |  |  |  |  | x | x | x | x | x | x | x | x | x | x |
| Blood for immune profile | x | x |  |  |  | x |  |  |  |  | x | x | x | x | x | x | x | x | x | x |
| Blood for flow cytometry |  | x | x | x | x | x | x | x | x | x | x | x | x | x | x | x | x | x | x | x |
| F-18 FDG PET/CT |  | x |  |  |  |  |  |  |  |  |  |  |  | x |  |  |  |  |  |  |
| Study medication |  |  |  |  |  |  |  |  |  |  |  |  |  |  |  |  |  |  |  |  |
| Bortezomib |  | x | x | x | x | x | x | x | x |  |  |  |  |  |  |  |  |  |  |  |
| Cyclophosphamide |  |  |  |  |  |  |  |  |  | x | x | x | x | x |  |  |  |  |  |  |

^a^ Urine pregnancy testing was only performed in sexually active women

^b^ If anti-HIV was negative on screening day and less than 1 week before enrollment, no repeat HIV testing was required

^c^ Blood chemistry included blood urea nitrogen, creatinine, liver function test, and fasting blood sugar (only if the patient had diabetes mellitus)

**Abbreviations:** CRP/ESR, C-reactive protein/erythrocyte sedimentation rate; D, day; F-18 FDG PET/CT, F-18 fluorodeoxyglucose positron emission tomography-computed tomography; HIV, human immunodeficiency virus; IFN-gamma, interferon gamma; W, week

| **Supplementary Table 2.** The number of opportunistic infections, the number of hospitalizations, and  the total hospital length of stay in days compared between 72 weeks before and 72 weeks after enrollment |
| --- |

|  | **72 weeks before BTZ initiation** | | | **72 weeks after BTZ initiation** | | |
| --- | --- | --- | --- | --- | --- | --- |
| **Case no.** | **OIs** | **Hospitalizations** | **Total LOS** | **OIs** | **Hospitalizations** | **Total LOS** |
| 1 | 2 | 5 | 75 | 3 | 6 | 114 |
| 2 | 2 | 2 | 40 | 3 | 4 | 75 |
| 3 | 3 | 4 | 126 | 3 | 4 | 87 |
| 4 | 1 | 6 | 109 | 1 | 2 | 35 |
| 5 | 1 | 3 | 68 | 0 | 2 | 20 |
| Total | 9 | 20 | 418 | 10 | 18 | 331 |
| Types of OIs | *M. abscessus* – 6 episodes from 5 patients  *M. scrofulaceum* – 1 episode  MAC – 1 episode  *T. marneffei* – 1 episode | | | *M. abscessus* – 6 episodes from 4 patients *T. marneffei* – 3 episodes from 3 patients  VZV – 1 episode | | |

| **Abbreviations:** BTZ, bortezomib; LOS, hospital length of stay; *M. abscessus*, *Mycobacterium* abscessus; *M.* *scrofulaceum*, *Mycobacterium*  *scrofulaceum*; MAC, Mycobacterium avium complex; OIs, opportunistic infections; *T. marneffei*, *Talaromyces*marneffei; VZV, varicella  zoster virus;  **Supplementary Table 3.** Results of F-18 PET/CT whole body scan at baseline and during the follow-up period | | | |
| --- | --- | --- | --- |
| **Case no.** | **Baseline PET/CT** | **Follow-up PET/CT ***  **(6 months to 2 years after study enrollment)** |  |
| **Case 1** | **At enrollment**   - Multiple lymphadenitis at right   lower paratracheal node, bilateral axillary nodes, bilateral para-aortic nodes, bilateral iliac nodes, and bilateral inguinal nodes | **At 6 months after enrollment**   - Slightly increased size of right lower   paratracheal node, slightly increased size and metabolism of bilateral axillary node, left para-aortic node, bilateral external iliac node, and bilateral inguinal nodes; new left internal mammillary node |  |
| **Case 2** | **At enrollment**   - Multiple hypermetabolic   mediastinal nodes at the pretracheal region, bilateral hilar region, prevascular space, and subcarina; few hypermetabolic nodes at bilateral inguinal region; hepatosplenomegaly | **At 6 months after enrollment**   - No significant change in size or   hypermetabolism of multiple mediastinal nodes or bilateral inguinal nodes; no change in hepatosplenomegaly |  |
| **Case 3** | **At enrollment**   - Bilateral hypermetabolic cervical   lymphadenitis (more prominent on the left), hypermetabolic submantle nodes, left retropharygeal node, left mediastinal nodes, bilateral hypermetabolic supraclavicular node, bilateral axillary nodes; mild hepatosplenomegaly; multiple hypermetabolic left para-aortic nodes and left external iliac node | **At 2 years after enrollment**   - Improvement in hypermetabolic   lymphadenopathies at left cervical, left axillary, bilateral retrocrural, para-aortic, and left iliac regions   - Multiple new hypermetabolic   lymphadenopathies at mediastinal, right axillary, right hilar, right iliac, and right inguinal regions   - Slightly increased uptake in generalized   edema of the right thigh   - Unchanged hepatosplenomegaly and   generalized increased metabolism in spleen, axial spine, and proximal bones of all extremities |  |
| **Case 4** | **At enrollment**   - Bilateral hypermetabolic enlarged   cervical nodes, bilateral enlarged supraclavicular nodes, multiple enlarged mediastinal nodes at right paratracheal region, bilateral enlarged axillary nodes, intramammary nodes, and pelvic nodes; matted left iliac node and bilateral inguinal nodes   - Skin thickening with generalized   slightly increased uptake at proximal left  thigh from inflammatory process | **At 1 year after enrollment**   - Decreased size of hypermetabolic   lymphadenitis at left cervical lymph nodes group III, left axilla, left intramammary, and left internal iliac regions   - New hypermetabolic lymphadenitis at   splenic hilum, and multiple right inguinal lymph nodes. Increased size of hypermetabolic lymphadenitis at left cervical nodes group I, right axilla, para-aortic, and right inguinal regions   - Progression of bilateral skin thickening   with slightly increased uptake at proximal bilateral thighs |  |
| **Case 5** | **Baseline**   - Increased uptake at multiple   lymphadenopathies at left cervical region (levels IIb and III), left superficial parotid lymph node, and right hilar lymph node; mild mucoperiosteal thickening at left maxillary sinus | **At 1 year after enrollment**   - Overall progression of active disease at   left cervical lymph node and left superficial parotid lymph node together with few left supraclavicular and right lower paratracheal lymphadenopathies |  |

*****The study protocol, which is described in Supplementary Table 1, shows that a PET/CT scan was scheduled to be performed at both study enrollment (baseline) and during 6 months to 1 year after study enrollment. Four of 5 study patients underwent follow-up PET/CT during 6 months to 1 year; however, 1 patient underwent follow-up PET/CT at 2 years after study enrollment due to the patient failing to appear for a scheduled follow-up scan within the 6-months to 1-year follow-up window.

**Abbreviations:** cm, centimeter; e.g., for example; F-18 FDG, F-18 fluorodeoxyglucose; PET/CT, positron emission tomography-computed tomography

**Supplementary Figure 1.** The gating strategy used to define B cell subsets. Total B cells (CD19+) were characterized as resting memory B cells (CD21+CD27+), tissue memory B cells (CD21-CD27-), naive B cells (CD21+CD27-), activated memory B cells (CD21-CD27+CD38-_low_), and ASC (CD21-CD27+CD38_high_).

**Supplementary Figure 2.** The gating strategy used to identify activated T cell subsets. CD4+ and CD8+ T cells were determined for cells that express CD38 and HLA-DR.

**Supplementary Figure 3.** Kinetic analysis of activated CD4+ and CD8+ T cells. Activated T cells were divided into 2 phenotypes, including cells that express HLA-DR without CD38 (CD38-DR+) and cells that express both CD38 and HLA-DR (CD38+DR+). The percentages of each phenotype of activated cells among CD4+ and CD8+ T cell populations were determined at different time points during the course of the study. One asterisk (*) indicates the time point when the patient was diagnosed with a proven opportunistic infection, and 2 asterisks (**) indicate the time point when the patient began receiving intravenous antibiotic therapy due to a worsening of clinical symptoms and signs.

**Supplementary Figure 4.** The effect of autoantibody level on the phosphorylation of STAT-1. The ability of plasma samples to inhibit the phosphorylation of the STAT-1 protein was compared among different time points when using an increased amount of IFN-γ (100 ng/mL). The percentage of cell population with pSTAT-1 expression and the mean fluorescence intensity of pSTAT-1 population were also compared among different time points.


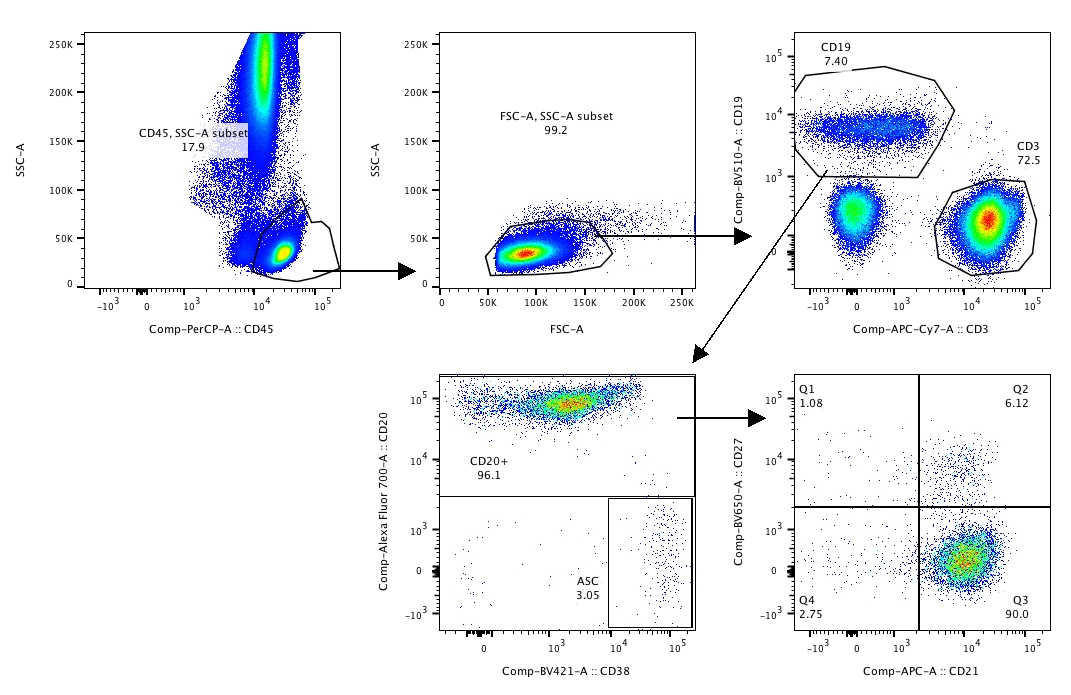


**Supplementary Figure 1.** The gating strategy used to define B cell subsets


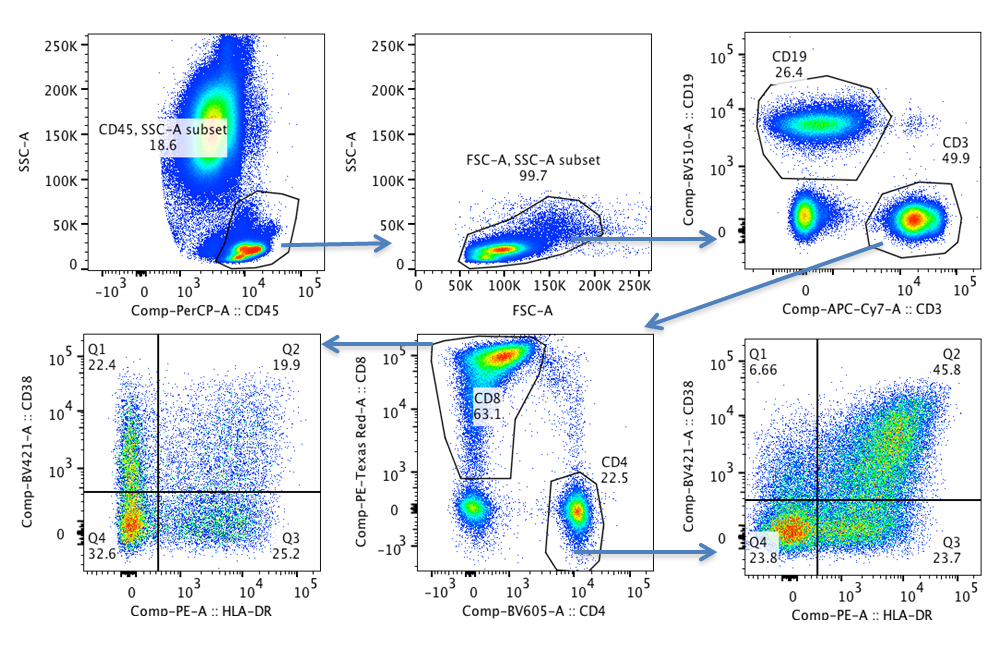


**Supplementary Figure 2.** The gating strategy used to identify activated T cell subsets


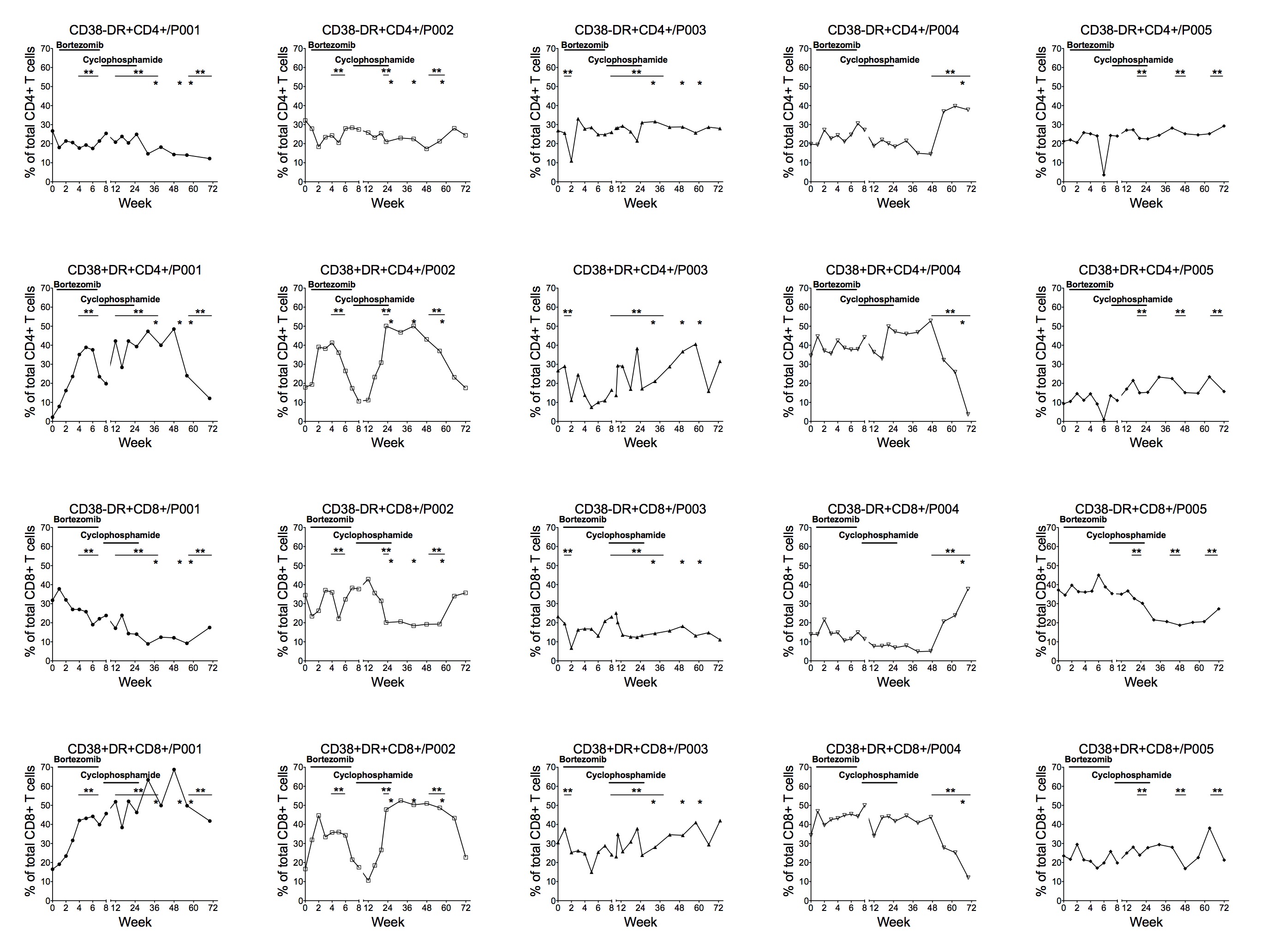


**Supplementary Figure 3.** Kinetic analysis of activated CD4+ and CD8+ T cells. One asterisk (*) indicates the time point when the patient was diagnosed with a proven opportunistic infection, and 2 asterisks (**) indicate the time point when the patient began receiving intravenous antibiotic therapy due to a worsening of clinical symptoms and signs. Columns 1-5 show the data for study patients 1-5 (P001-P005), respectively.


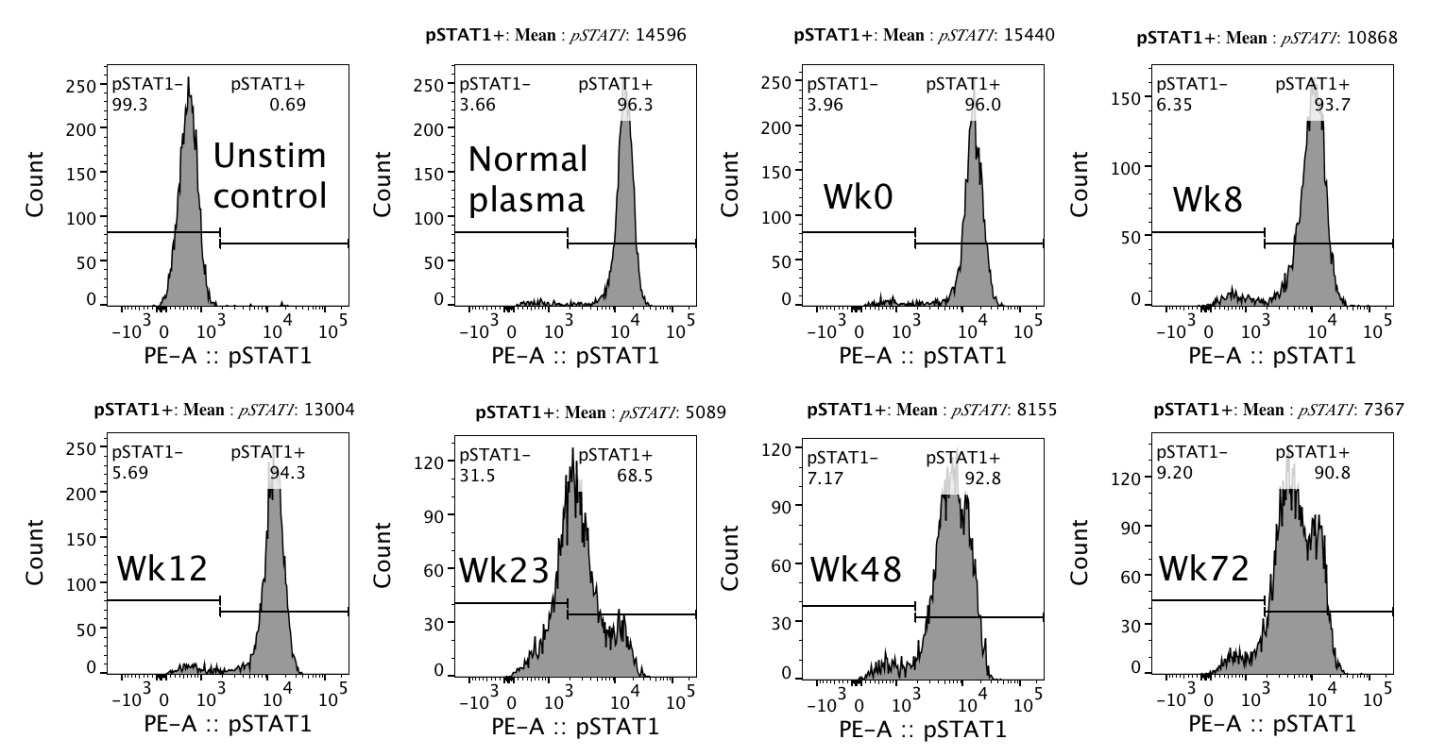


**Supplementary Figure 4.** The effect of autoantibody level on the phosphorylation of STAT-1
